# Rhein alleviates hepatic steatosis in NAFLD mice by activating the AMPK/ACC/SREBP1 pathway to enhance lipid metabolism

**DOI:** 10.1186/s10020-025-01304-4

**Published:** 2025-07-10

**Authors:** Weiwei Dai, Qishu Hou, Jifeng Ye

**Affiliations:** Department of Pharmacy, The Second Affiliated Hospital and Yuying Children’s Hospital of Wenzhou Medical University, No. 109 West Road, Lucheng District, Wenzhou, 325000 Zhejiang China

**Keywords:** Rhein, Non-alcoholic fatty liver disease, 5'-adenosine monophosphate-activated protein kinase signaling pathway, Acetyl-coa carboxylase, Sterol regulatory element-binding protein 1

## Abstract

**Background:**

Non-alcoholic fatty liver disease (NAFLD) is a common metabolic liver disorder characterized by excessive lipid accumulation. The 5’-adenosine monophosphate-activated protein kinase (AMPK)/acetyl-CoA carboxylase (ACC)/sterol regulatory element-binding protein 1 (SREBP1) pathway plays a pivotal role in regulating lipid metabolism. Rhein, a natural compound, has demonstrated hepatoprotective potential; however, its mechanism of action in NAFLD remains unclear. This study aimed to investigate whether rhein ameliorates NAFLD through modulation of the AMPK/ACC/SREBP1 pathway.

**Methods:**

A murine NAFLD model was established using a high-fat diet (HFD). Mice were treated with varying doses of rhein, and their body weight, liver, kidney, and retroperitoneal fat weights were recorded. Liver pathology was assessed by histological examination and Oil Red O staining. Serum lipid profiles, liver function biomarkers, and inflammatory cytokine levels were measured. Western blotting was employed to analyze the expression and phosphorylation of AMPK pathway-related proteins (AMPK, ACC, and SREBP1). To validate the involvement of this pathway, AMPK-IN-3 was intraperitoneally administered in combination with high-dose rhein to a subset of HFD-fed mice.

**Results:**

Rhein treatment significantly reduced body weight gain, organ weights, hepatic lipid accumulation, serum cholesterol and triglyceride levels, and the expression of inflammatory cytokines in NAFLD mice. It also improved liver function markers, enhanced AMPK phosphorylation, promoted ACC phosphorylation, and inhibited SREBP1 expression. Notably, co-treatment with AMPK-IN-3 attenuated these beneficial effects, confirming the mechanistic involvement of the AMPK/ACC/SREBP1 pathway.

**Conclusion:**

Rhein confers protective effects against HFD-induced NAFLD by activating the AMPK/ACC/SREBP1 signaling pathway, thereby enhancing hepatic lipid metabolism, reducing steatosis, and alleviating liver injury and inflammation. These findings suggest that rhein may serve as a promising therapeutic candidate for NAFLD.

**Supplementary Information:**

The online version contains supplementary material available at 10.1186/s10020-025-01304-4.

## Introduction

Non-alcoholic fatty liver disease (NAFLD) is a complex metabolic disorder that encompasses a spectrum of clinical, histological, and pathophysiological stages, ranging from mild steatosis to liver fibrosis and non-alcoholic steatohepatitis. This condition may further progress to severe outcomes, including liver failure, cirrhosis, and hepatocellular carcinoma (Perakakis et al. 2020). NAFLD is characterized by hepatic fat accumulation in the absence of other known causes, such as excessive alcohol intake, chronic medication use, or hereditary metabolic disorders (Cataldo et al. 2021). With the global rise in metabolic syndrome, obesity, and diabetes, the incidence of NAFLD has increased sharply, impacting approximately one-fourth of the global population (Guo et al. 2022). Currently, there are no clinically approved pharmacological therapies for NAFLD; current management primarily relies on lifestyle modifications involving diet and exercise. However, many patients often struggle to adhere to these long-term modifications (Guo et al. 2022). Therefore, exploring the pathogenesis of NAFLD and identifying potential therapeutic targets are crucial for drug development.

In recent years, research into the underlying molecular and cellular mechanisms contributing to NAFLD development has deepened significantly, uncovering the crucial involvement of numerous key signaling pathways in disease progression. Among these, the 5’-adenosine monophosphate-activated protein kinase (AMPK)/sterol regulatory element-binding protein 1 (SREBP1)/acetyl-CoA carboxylase (ACC) signaling pathway has attracted considerable attention (Ye et al. 2022). AMPK functions as a ubiquitous energy and nutrient sensor in eukaryotic cells, playing a central role in maintaining cellular energy homeostasis and regulating multiple aspects of cellular metabolism (Cui et al. 2023). ACC, a pivotal enzyme in fatty acid synthesis and metabolism regulation, has emerged as a promising therapeutic target in a range of diseases, including NAFLD, cancer, bacterial infections, and diabetes mellitus (Wu and Huang 2020). Moreover, SREBP1 serves as a crucial transcriptional regulator of lipid metabolism and has been demonstrated to modulate macrophage activation states (Oishi et al. 2023). Studies have demonstrated that under energy-deficient conditions, AMPK is activated and directly phosphorylates ACC, thereby inhibiting its enzymatic activity, reducing malonyl-CoA production, and consequently suppressing fatty acid synthesis. Simultaneously, AMPK indirectly downregulates the expression of downstream lipogenic enzymes (such as FASN, SCD1) by inhibiting SREBP1 expression and its precursor cleavage activation, further attenuating hepatic lipogenesis (Park et al. 2019; Li et al. 2022). Therefore, the AMPK→ACC→SREBP1 axis represents a classical linear pathway for energy sensing and lipid metabolic regulation, and alterations in this pathway may play a pivotal role in the pathological progression of NAFLD.

Rhein, a representative anthraquinone compound (Pei et al. 2021), has demonstrated unique advantages in the treatment of NAFLD. Derived from multiple botanical sources, rhein can be extracted from various traditional Chinese medicinal herbs such as *Polygonum multiflorum*, *Cinnamomum cassia*, *Polygonum cuspidatum*, and *Rheum palmatum* (Li et al. 2021). Preclinical studies have shown that rhein-lysine improves liver function by reducing hepatic lipid accumulation and downregulating inflammatory cytokine expression (Wei et al. 2016). Furthermore, rhein has been found to ameliorate NAFLD and related conditions through mechanisms involving liver X receptor-mediated energy balance, metabolic regulatory pathways, and immune-modulatory effects during hepatic steatosis (Sheng et al. 2011). These findings suggest that rhein may exert liver-specific protective effects in NAFLD treatment. In the context of metabolic syndrome, rhein has been shown to alleviate hyperglycemia-induced oxidative stress and apoptosis, and protect against mitochondrial dysfunction by activating the AMPK/Sirt1/PGC-1α signaling cascade (Liu et al. 2023). In terms of immune regulation, rhein can selectively influence macrophage polarization, suppressing pro-inflammatory M1 polarization while promoting anti-inflammatory M2 polarization. This effect is achieved through a novel mechanism involving the NFATc1/Trem2 axis, thereby contributing to immune homeostasis (Li et al. 2023). Despite these promising findings, the direct mechanistic interaction between rhein and the AMPK/ACC/SREBP1 pathway in NAFLD has not yet been fully elucidated. Therefore, the present study aims to establish a murine model of NAFLD to evaluate changes in hepatic function and lipid metabolism following rhein treatment, and to explore the regulatory mechanism of rhein via the AMPK/ACC/SREBP1 signaling pathway. From an innovation standpoint, this study is the first to systematically validate the role of rhein in modulating hepatic lipid metabolism through the AMPK/ACC/SREBP1 pathway in the context of NAFLD, thereby providing a potential new candidate for NAFLD therapy.

## Materials and methods

### Ethical statement

All experimental animals in this study were used exclusively for medical research purposes. All procedures were carried out after discussion and approval by the Animal Committee of The Second Affiliated Hospital and Yuying Children's Hospital of Wenzhou Medical University. All operations strictly adhered to the regulations for the management of laboratory animals.

### Animal experiment and grouping

A total of 56 six-week-old specific pathogen free male C57BL/6J mice (weighing 18–22 g) were available from Shanghai Model Organisms Center, Inc. The mice were acclimatized for one week under standard laboratory conditions: constant temperature (20–26 °C), relative humidity (40–70%), a natural light/dark cycles, and adequate air circulation before the experimental procedures were initiated.

To establish the NAFLD model, all mice except those in the normal group were fed a high-fat diet (HFD) for 8 consecutive weeks. The HFD (Catalog No.: D12492, Research Diets, Inc., New Jersey, USA) consisted of 40% fat, 20% protein, and 40% carbohydrates by caloric composition. The normal group was maintained on a standard laboratory diet (12% fat, 23% protein, 65% carbohydrates). Successful NAFLD modeling was confirmed through increased body weight, elevated serum alanine aminotransferase (ALT) and aspartate aminotransferase (AST) levels, and histopathological assessment of liver tissues using Hematoxylin and Eosin (H&E) and Oil Red O staining. Mice were randomly divided into 10 groups (*n* = 8 per group). Randomization was accomplished using a random number table generated by Excel, and the entire process was carried out by independent researchers who did not participate in subsequent experimental operations to avoid bias due to human intervention. The groups were as follows: Normal group (regular diet); Rhein group (150 mg/kg) (regular diet + high-dose Rhein group (150 mg/kg); NAFLD group; NAFLD + Low Rhein group (25 mg/kg); NAFLD + Mild Rhein group (50 mg/kg); NAFLD + High Rhein group (150 mg/kg); NAFLD + dimethyl sulfoxide (DMSO) group (1%, vehicle control, intraperitoneal injection); NAFLD + AMPK-IN-3 group (10 mg/kg); NAFLD + High-Rhein + DMSO group; and NAFLD + High-Rhein + AMPK-IN-3 group group (10 mg/kg). Rhein (Rhein, R7269, Sigma, purity ≥ 98%, HPLC) was dissolved in phosphate-buffered saline and administered via oral gavage once every 4 days for 8 weeks. The dosage was determined based on previous animal studies and preliminary pilot experiments (Wei et al. 2016; Sheng et al. 2011), designed to cover a pharmacologically relevant dose range. The dosing interval (once every four days) was chosen to maintain pharmacokinetic stability while minimizing animal stress and interference with normal feeding behavior. The AMPK inhibitor AMPK-IN-3 (HY-151361, MCE) was dissolved in 1% DMSO (Sigma, 34869) and administered intraperitoneally at 10 mg/kg, also once every 4 days for 8 weeks. Body weight was recorded before each administration. At the end of the experiment, peripheral blood, liver, kidneys, and retroperitoneal fat pads were collected for subsequent analysis (Wei et al. 2016; Sheng et al. 2011, 2019).

### Serum biochemical analysis

Following HFD feeding and rhein treatment, mice were anesthetized, and 1 mL of blood was gathered from the abdominal aorta into anticoagulant-free tubes. Samples were centrifuged at 3000 rpm for 15 min at 4 °C to acquire the serum. Serum levels of AST, triglycerides (TG), ALT, high-density lipoprotein cholesterol (HDL-C), and low-density lipoprotein cholesterol (LDL-C) were determined usinga Hitachi 7020 Automatic Clinical Analyzer (Tokyo, Japan) (Sheng et al. 2019; Wu et al. 2022).

### Enzyme-linked immunosorbent assay (ELISA) of inflammatory cytokine levels

Following HFD and rhein treatment, serum samples were gathered to assess inflammatory cytokine secretion. Serum levels of interleukin-6 (IL-6) and tumor necrosis factor-α (TNF-α) were assessed using commercia ELISA kits (TNF-α: BMS607, Thermo; IL-6: 88–7064-88, eBioscience). Absorbance was read at 450 nm and 570 nm using a SpectraMax^®^i3 spectrophotometer. To account for background interference, absorbance at 570 nm was subtracted from that at 450 nm. Cytokine concentrations were quantified based on the standard curve generated in each assay (Wu et al. 2022).

### HE staining

After euthanizing the mice, liver tissues were harvested and fixed in 4% paraformaldehyde. Tissues were embedded in paraffin and sectioned at a thickness of 3 μm. Sections were then dewaxed, rehydrated, and stained using an HE staining kit (G1121, Solarbio). After mounting with neutral resin, the stained sections were examined under a Zeiss optical microscope (Zeiss AG, Oberkochen, Germany) at 200× magnification to evaluate liver histopathological changes (Fang et al. 2023).

### Oil red O staining

For lipid deposition assessment, fresh liver tissues were embedded in optimal cutting temperature (OCT) compound and frozen. Frozen liver sections (5 μm thick) were prepared and stained with Oil Red O (O8010, Solarbio). Prior to staining, sections were immersed in anhydrous propylene glycol for 5 min to prevent water contamination. A 0.5% w/v Oil Red O solution, pre-dissolved in isopropanol and cooled, was used to stain the sections for 10 min. Sections were then differentiated in 85% propylene glycol for 5 min, rinsed, and mounted with glycerol gelatin. Lipid droplets were observed under a Zeiss optical microscope at 200× magnification (Fang et al. 2023; Lee et al. 2023).

### Western blotting (WB)

Protein were extracted from liver tissues using RIPA lysis buffer supplemented with PMSF (Catalog No.: P0013C, Beyotime, Shanghai, China). After incubation, the lysates were centrifuged to collect the supernatant. Total protein concentration was quantified using a BCA assay kit (Catalog No.: 23227, ThermoFisher, USA). Equal amounts of protein (50 µg) were mixed with 2× SDS loading buffer, boiled, and then separated by electrophoresis on a 10% SDS-PAGE gel (Catalog No.: G2177-50T, Servicebio, Wuhan, China). Proteins were transferred onto PVDF membranes (Catalog No.: ISEQ00010, Millipore). Membranes were blocked with 5% skimmed milk for 1 h at room temperature to prevent non-specific binding and incubated overnight at 4 °C with primary antibodies: AMPKα1 (1:2000, ab32047, Abcam, Cambridge, UK), p-AMPKα1 (1:2000, ab131357, Abcam), p-ACC (1:1000, #11818, CST, USA), ACC (1:1000, #3676, CST, USA), SREBP1 (1:2000, ab313881, Abcam), and GAPDH (ab9485, 1:2500, Abcam) as an internal reference. After being washed with TBST, membranes were incubated with HRP-conjugated secondary antibody, specifically a (goat anti-rabbit IgG H&L, ab97051, 1:2000, Abcam) for 1 h. After that, the membranes were washed and developed using ECL reagent (Catalog No.: abs920, Abxin Biotechnology Co., Ltd., Shanghai, China), prepared by mixing equal volumes of solutions A and B in the dark. Images were captured with a Bio-Rad imaging system (Bio-RAD, USA), and band intensities were quantified using Quantity One software version 4.6.2. Protein expression levels were normalized to GAPDH and expressed as the ratio of gray values. Each experiment was repeated three times, and average values were reported (Lee et al. 2023; Ma et al. 2021; Ahuja et al. 2022; Lin et al. 2022; Zeng et al. 2022).

### Drug-ingredient-target-disease network construction

Active compounds of rhein were retrieved from the TCMSP database (https://old.tcmsp-e.com/tcmsp.php), applying screening thresholds of oral bioavailability > 30% and drug-likeness > 0.18. Target genes corresponding to these compounds were annotated using the UniProt database (https://www.uniprot.org/). NAFLD-related genes were identified from the GeneCards database using “Non-alcoholic fatty liver disease” as the search term. The intersection between drug targets and disease-associated genes was used to build the network. This information was imported into Cytoscape software (version 3.8.0) to construct a drug-ingredient-target-disease network (Liu et al. 2020).

### Kyoto encyclopedia of genes and genomes (KEGG) pathway enrichment analysis

Candidate target genes were subjected to KEGG pathway enrichment using the “ClusterProfiler” package (http://www.bioconductor.org/packages/ClusterProfiler/) in R language, with *P*-value < 0.05 setting as the threshold for significant enrichment. The top enriched signaling pathways were visualized using the “ggplot2” package (http://www.bioconductor.org/packages/ggplot2/) (Zhong et al. 2023).

### Statistical analysis

All data were analyzed using SPSS statistical software version 24.0 (SPSS, Inc., USA). Normality and homogeneity of variances were assessed. Normally distributed measurement data were presented as mean ± standard deviation. For comparisons between two groups, independent-sample t-tests were utilized, while for multiple-group comparisons, one-way analysis of variance with Tukey’s post hoc test was employed. Statistical significance was considered at a *P*-value < 0.05 (De et al. 2024).

## Results

### Rhein ameliorates lipid accumulation in HFD-induced NAFLD mice

**Fig. 1:**
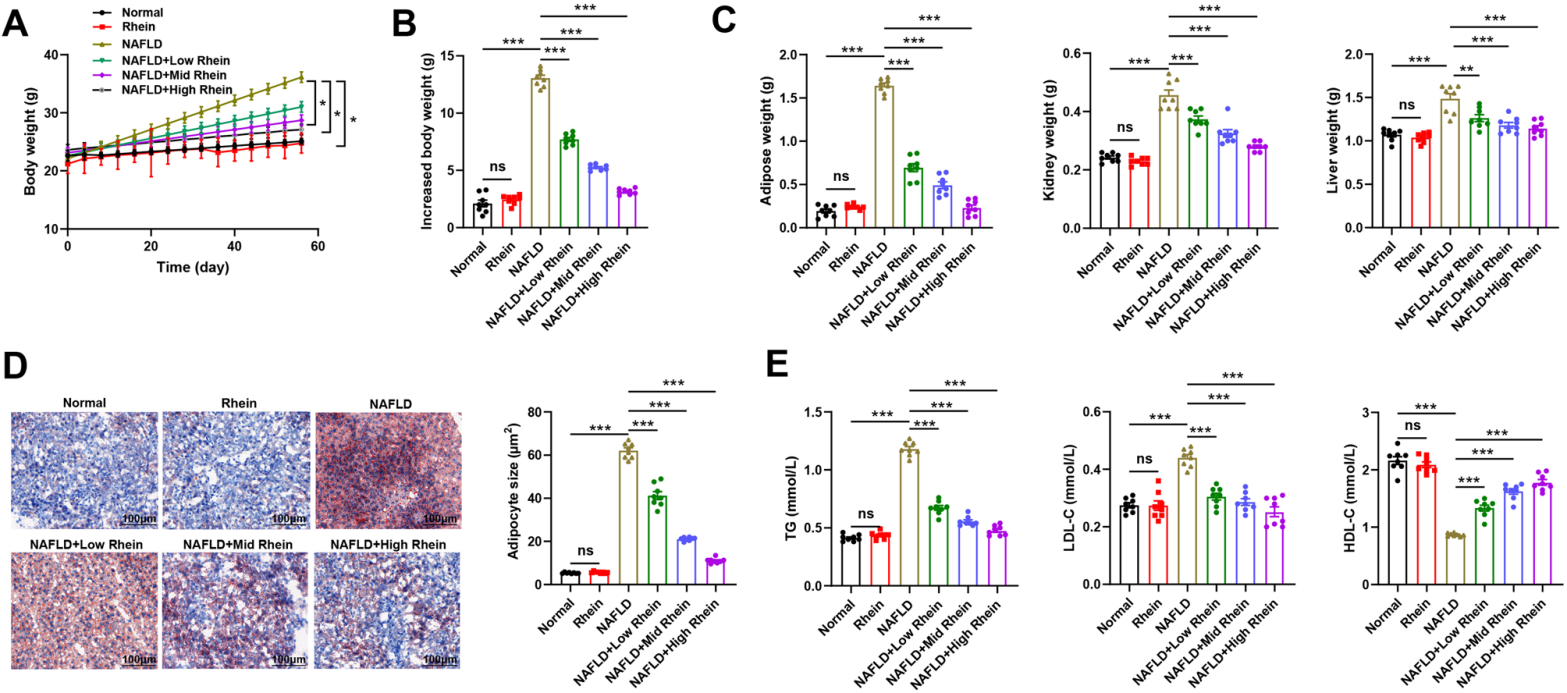
Rhein ameliorates lipid accumulation in HFD-induced NAFLD mice. Note: **A** Changes in body weight during high-fat feeding and administration of different doses of rhein; **B** Magnitude of body weight gain after eight weeks of high-fat feeding and administration of different doses of rhein; **C** Weights of the kidneys, livers, and retroperitoneal fat pads at the end of the feeding period; **D** Oil Red O staining to detect lipid droplet formation in mouse livers, bar = 100 μm; **E** Automatic biochemical analyzer detection of cholesterol and triglyceride-related indicators (TG, HDL-C, and LDL-C) levels in mouse peripheral blood serum; *n* = 8, data are presented as mean ± SD, ns indicates no significant difference between the two groups, * indicates *P* < 0.05 when comparing between two groups, *** represents *P* < 0.001 between the two groups

To ascertain the effect of rhein on lipid accumulation in NAFLD mice induced by an HFD, we established a murine NAFLD model and administered rhein for eight consecutive weeks. The results disclosed that compared with the normal group, mice in the NAFLD group exhibited progressive weight gain over time, while rhein administration mitigated this trend, particularly in the high-dose group (Fig. 1 A). By the end of the intervention, body weight gain was significantly reduced in all rhein-treated groups relative to the NAFLD group, with the most notable effect observed in the high-dose group (Fig. 1B). Additionally, rhein reduced the weights of the kidneys, liver, and retroperitoneal fat pads in NAFLD mice, again showing a dose-dependent effect (Fig. 1 C). Histological analysis revealed reduced hepatic lipid droplet accumulation in the rhein-treated groups, with the high-dose group showing the greatest improvement (Fig. 1D). In contrast with the normal group, mice in the NAFLD group displayed elevated serum levels of LDL-C and TG and decreased HDL-C levels. Rhein intervention diminished TG and LDL-C levels and enhanced HDL-C levels, with the high-dose group demonstrating the most pronounced effect (Fig. 1E). Additionally, no significant differences were observed in the Rhein group (normal diet + Rhein) in terms of body weight, adipose organ indices, blood lipid levels, hepatic histology, and inflammatory cytokine expression, suggesting that rhein does not exert adverse effects on metabolic parameters under non-stressed conditions (Fig. 1).

These results indicate that rhein effectively alleviates HFD-induced lipid accumulation and improves fat metabolism.

### Rhein alleviates liver injury and inflammation in NAFLD mice

**Fig. 2:**
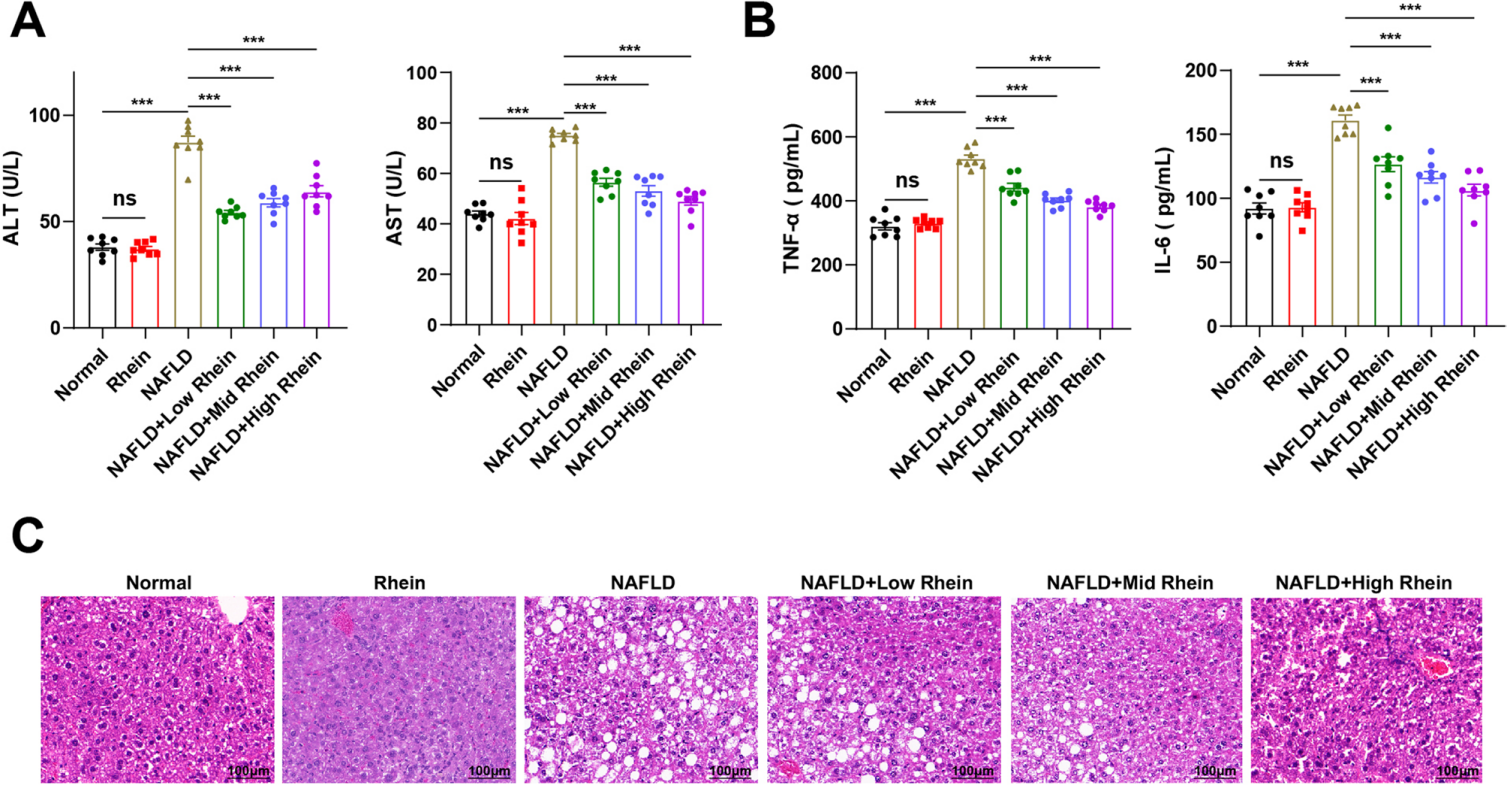
Rhein alleviates liver injury and inflammation in NAFLD mice. Note: **A** Automatic biochemical analyzer detection of liver function-related indicators ALT and AST levels in mouse peripheral blood serum; **B** ELISA detection of the secretion levels of inflammatory cytokines TNF-α and IL-6 in serum; **C** HE staining to detect mouse liver pathology, bar = 100 μm; *n* = 8, data are presented as mean ± SD, ns indicates no significant difference between the two groups, *** represents *P* < 0.001 between the two groups

To elucidate the ameliorative effects of rhein on liver injury and inflammation in NAFLD mice, we assessed hepatic function and inflammatory responses following rhein treatment. The results uncovered (Fig. 2 A) that compared with the normal group, mice in the NAFLD group demonstrated elevated serum levels of AST and ALT, suggesting that HFD caused significant liver injury, while rhein intervention reduced serum ALT and AST levels, with a more pronounced effect observed in the high-dose group. In terms of inflammatory markers, in comparison with the normal group, mice in the NAFLD group exhibited enhanced serum levels of TNF-α and IL-6, while rhein intervention decreased the levels of these pro-inflammatory cytokines, with a more significant effect in the high-dose group, implying its inhibitory effect on liver inflammation (Fig. 2B). Additionally, Histological analysis revealed that hepatocytes from NAFLD mice showed vacuolar degeneration and cytoplasmic changes, while rhein treatment led to substantial improvements in liver tissue structure, with a more prominent effect at higher doses (Fig. 2 C). Moreover, the Rhein group showed no significant differences from the normal group in terms of ALT/AST levels, inflammatory cytokines, or liver histology (*P* > 0.05), revealing that rhein does not cause hepatic toxicity under physiological conditions (Fig. 2).

In brief, rhein alleviates liver injury in NAFLD mice by improving liver function and inhibiting inflammation.

### Construction of drug-ingredient-target network

**Fig. 3:**
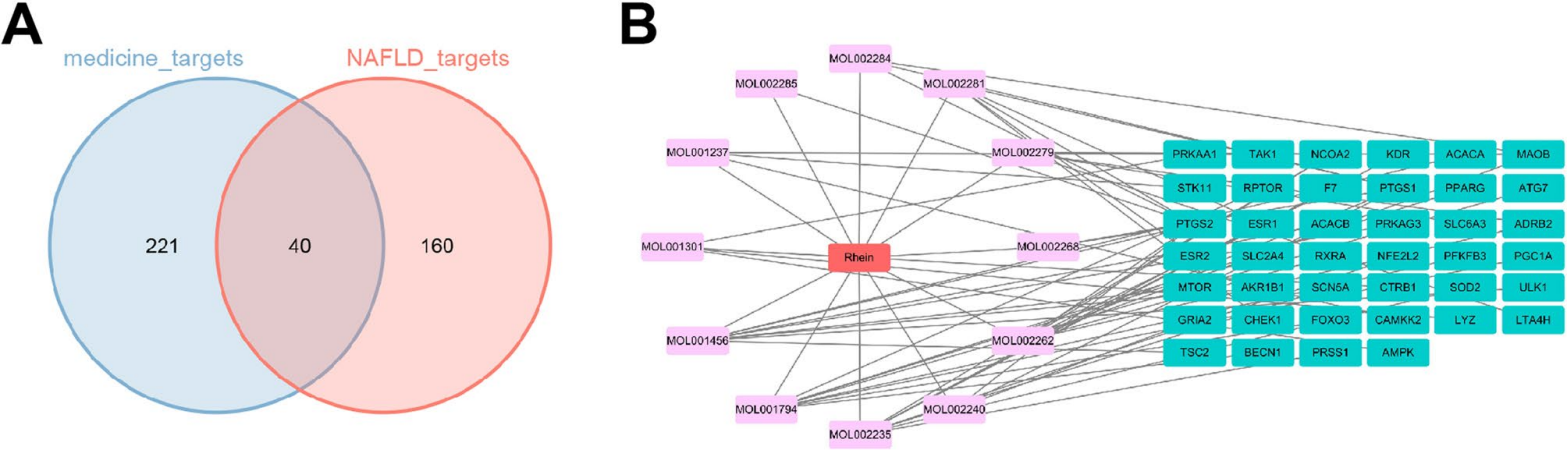
Construction of drug-ingredient-target network. Note: **A** Venn diagram showing the intersection of rhein’s targets and NAFLD-related genes; **B** Network diagram constructed based on drug-ingredient-target

To obtain the potential targets of rhein in alleviating liver injury in NAFLD mice, a total of 261 rhein-related targets were retrieved from the TCSMP database (Supplementary Table 1). In parallel, 200 NAFLD-related genes were obtained from the GeneCards database using “Non-alcoholic fatty liver disease” as the search keyword. By intersecting these genes, 40 overlapping genes were identified as candidate targets (Supplementary Table 2, Fig. 3 A). Based on these targets, we employed Cytoscape software to draw a “drug-ingredient-target” network network (Fig. 3B).

### Candidate genes of Rhein are enriched in the AMPK signaling pathway

**Fig. 4:**
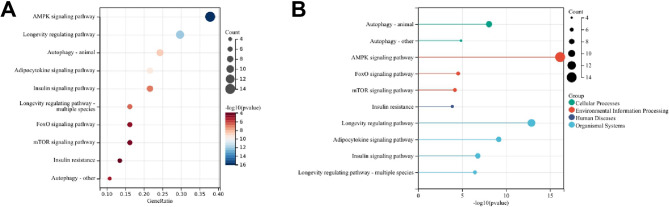
KEGG enrichment analysis of potential target genes. Note: **A** KEGG enrichment analysis bubble chart; **B** KEGG enrichment analysis bar chart

A total of 56 candidate genes potentially regulated by rhein were identified. To explore key regulatory pathways, KEGG enrichment analysis was performed on these targets. The results indicated significant enrichment in the AMPK signaling pathway (Fig. 4A-B). Based on these findings, we hypothesized that activation of the AMPK pathway may play an essential role in rhein’s protective effects against liver injury in NAFLD.

### Rhein promotes ACC phosphorylation and suppresses SREBP1 expression by activating the AMPK signaling pathway

**Fig. 5:**
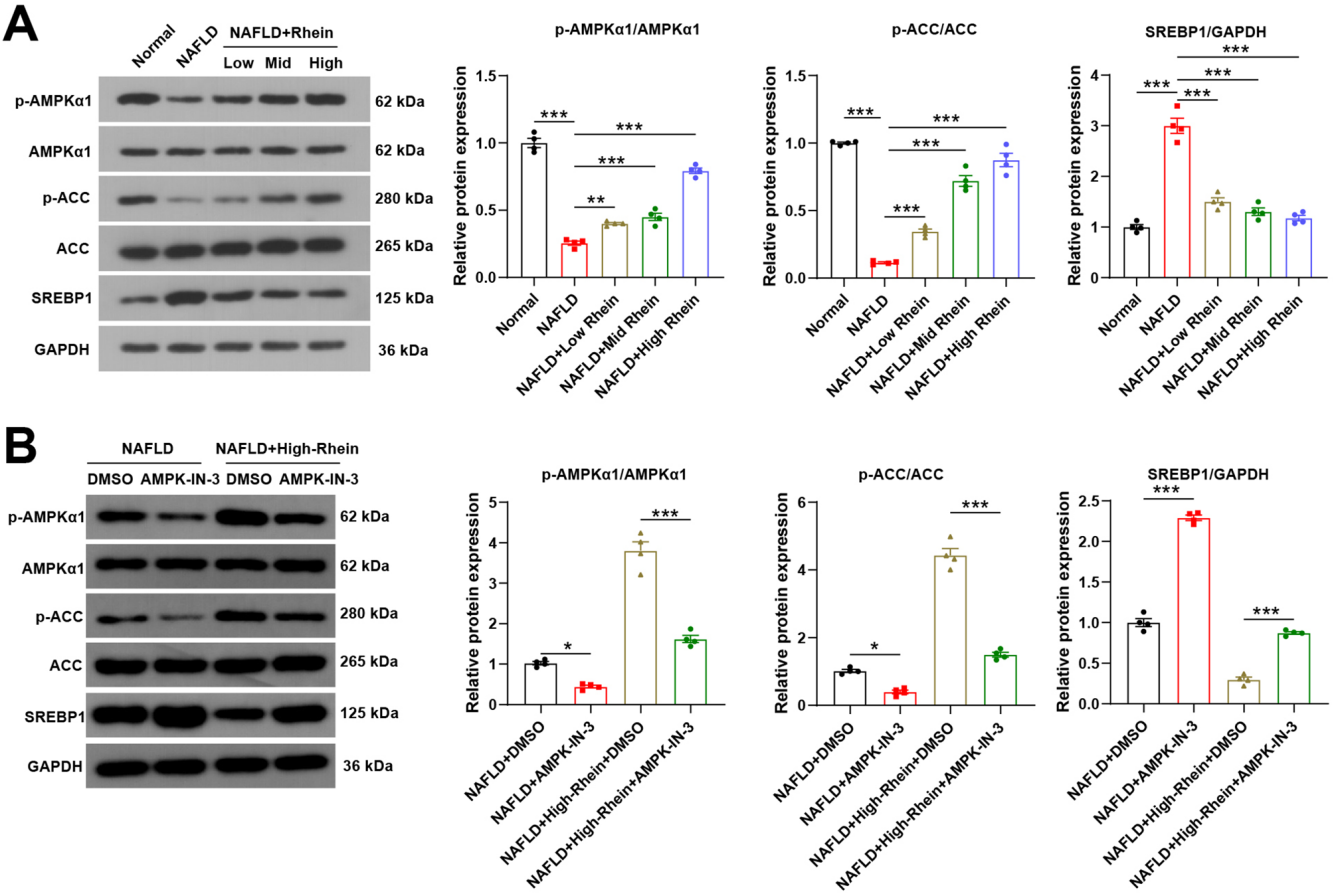
Rhein promotes ACC phosphorylation and suppresses SREBP1 expression by activating the AMPK signaling pathway. Note: **A** WB detection of the expression of related proteins in the AMPK signaling pathway, ACC phosphorylation levels, and SREBP1 expression levels in liver tissue of mice after eight weeks of high-fat feeding and administration of different doses of rhein; **B** WB detection of the expression of related proteins in the AMPK signaling pathway, ACC phosphorylation levels, and SREBP1 expression levels in liver tissue of mice after high-fat feeding, administration of high-dose rhein, and intraperitoneal injection of the AMPK signaling pathway inhibitor AMPK-IN-3; *n* = 4, data are presented as mean ± SD, * indicates *P* < 0.05 when comparing between two groups

Studies have shown that AMPK can affect liver lipogenesis by regulating ACC phosphorylation and SREBP1 expression (Park et al. 2019; Li et al. 2022). To verify this mechanism, we examined the phosphorylation levels of AMPKα1 and ACC, along with SREBP1 expression, in liver tissues from NAFLD mice treated with rhein. The results disclosed that compared to the normal group, NAFLD mice exhibited decreased phosphorylation of AMPKα1 and ACC and elevated SREBP1 expression levels. However, after rhein intervention, the phosphorylation levels of AMPKα1 and ACC elevated, and SREBP1 expression levels diminished, with a more significant effect observed in the high-dose group, suggesting that rhein could activate the AMPK signaling pathway, facilitate ACC phosphorylation, and reduce SREBP1 expression, thereby regulating lipid metabolism (Fig. 5 A).

To determine whether these regulatory effects are AMPK-dependent, an AMPK-IN-3 intervention group was established. Comparison between the NAFLD + DMSO group and the AMPK-IN-3 group revealed that AMPK-IN-3 further decreased p-AMPKα1 and p-ACC levels and upregulated SREBP1 expression, indicating suppression of AMPK activity under NAFLD conditions (Fig. 5B).

Notably, while high-dose rhein alone increased p-AMPKα1 and p-ACC and reduced SREBP1 levels, co-administration with AMPK-IN-3 markedly weakened or reversed these effects. This suggests that rhein’s modulation of ACC and SREBP1 is dependent on AMPK pathway activation (Fig. 5B).

These results imply that rhein alleviates hepatic lipid accumulation in NAFLD mice by activating the AMPK signaling pathway, thereby enhancing ACC phosphorylation and downregulating SREBP1 expression.

### Rhein improves lipid accumulation in NAFLD mice by activating the AMPK pathway

To determine whether the improvement of lipid accumulation in NAFLD by Rhein is mediated through the AMPK signaling pathway, four experimental groups were established: NAFLD + DMSO, NAFLD + AMPK-IN-3, NAFLD + High-Rhein + DMSO, and NAFLD + High-Rhein + AMPK-IN-3, to evaluate the impact of AMPK inhibition on the metabolic benefits of Rhein.

**Fig. 6:**
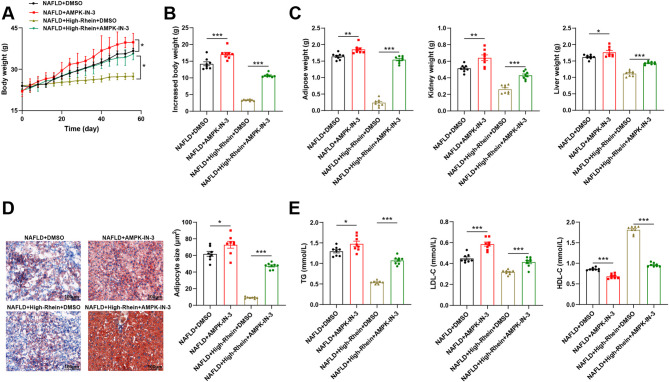
Rhein improves lipid accumulation in NAFLD mice by activating the AMPK pathway. Note: **A** Changes in body weight of mice during high-fat feeding, administration of high-dose rhein, and intraperitoneal injection of the AMPK signaling pathway inhibitor AMPK-IN-3; **B** Magnitude of body weight gain after feeding; **C** Weights of the kidneys, liver, and retroperitoneal fat pads after feeding; **D** Oil Red O staining to detect liver lipid droplet formation in mice, bar = 100 μm; **E** Automatic biochemical analyzer detection of cholesterol and triglyceride-related indicators TG, HDL-C, and LDL-C levels in mouse peripheral blood serum; *n* = 8, data are presented as mean ± SD, * indicates *P* < 0.05 when comparing between two groups, **P < 0.01, ***P < 0.001

As shown in Fig. 6A-B, the body weight gain in the NAFLD + AMPK-IN-3 group was higher than that in the NAFLD + DMSO group (*P* < 0.001), suggesting that suppression of AMPK activity aggravated NAFLD-related metabolic disturbances. In contrast, the NAFLD + High-Rhein + DMSO group exhibited a lower body weight, supporting the lipid-lowering effect of rhein (*P* < 0.001). However, in the NAFLD + High-Rhein + AMPK-IN-3 group, this effect was markedly attenuated, with a rebound in body weight (*P* < 0.001), indicating that AMPK inhibition weakened the effect of Rhein.

Organ mass analysis (Fig. 6 C) revealed that compared with the NAFLD + DMSO group, AMPK-IN-3 treatment further increased the weights of retroperitoneal fat, liver, and kidneys. Rhein treatment effectively reduced these organ weights, but its protective effects were partially reversed when co-administered with AMPK-IN-3.

Oil Red O staining (Fig. 6D) showed extensive lipid droplet accumulation and pronounced vacuolar degeneration in the AMPK-IN-3 group. In contrast, rhein treatment reduced lipid droplet accumulation and decreased the area of adipocytes, while the co-treatment with AMPK-IN-3 significantly attenuated these improvements (*P* < 0.001).

In terms of blood lipid levels (Fig. 6E), compared with the NAFLD + DMSO group, AMPK-IN-3 treatment increased the levels of TG and LDL-C, while decreasing HDL-C (*P* < 0.001). In contrast, rhein intervention showed a trend of decreasing TG and LDL-C levels and increasing HDL-C levels. However, in the NAFLD + High-Rhein + AMPK-IN-3 group, the regulatory effect of rhein on blood lipids was attenuated.

Taken together, these findings confirm that the AMPK signaling pathway plays a key role in mediating the beneficial metabolic effects of rhein. The reversal of these effects by AMPK-IN-3 suggests that rhein’s action in improving lipid metabolism in NAFLD is AMPK-dependent.

### Rhein alleviates liver injury and inflammation in NAFLD mice by activating the AMPK pathway

**Fig. 7:**
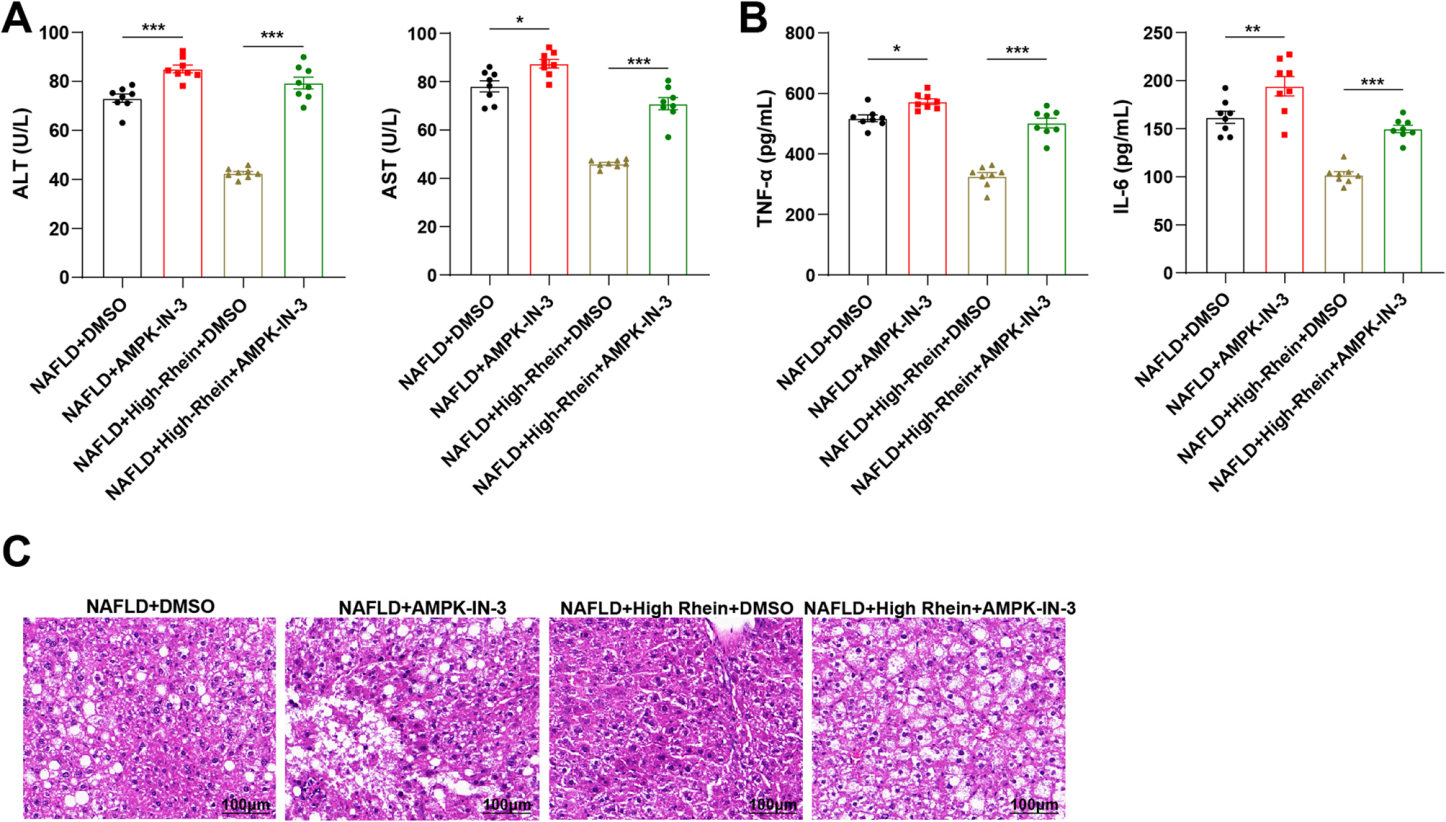
Rhein alleviates liver injury and inflammation in NAFLD mice by activating the AMPK pathway. Note: **A** Liver function-related indicators ALT and AST levels in peripheral blood serum of mice were detected using an automatic biochemical analyzer; **B** ELISA was used to detect the secretion levels of inflammatory cytokines TNF-α and IL-6 in serum; **C** Pathological conditions of mouse livers were examined using HE staining, bar = 100 μm; *n* = 8, data are presented as mean ± SD, and * indicates *P* < 0.05 when comparing between the two groups, **P < 0.01, ***P < 0.001

To further clarify the role of the AMPK pathway in the hepatoprotective and anti-inflammatory effects of rhein, we evaluated liver injury and inflammation across the same treatment groups. serum ALT and AST levels were significantly higher in the NAFLD + AMPK-IN-3 group compared to the NAFLD + DMSO group (*P* < 0.001), indicating aggravated liver damage following AMPK inhibition. Rhein administration markedly reduced ALT and AST levels, reflecting improved liver function. However, when combined with AMPK-IN-3, this effect was significantly reduced (*P* < 0.001), further indicating that rhein’s hepatoprotective action relies on AMPK activation (Fig. 7 A), suggesting that the effect of Rhein depends on the AMPK pathway.

The results of inflammatory cytokine analysis (Fig. 7B) further supported this conclusion: the levels of TNF-α and IL-6 in the NAFLD + AMPK-IN-3 group were higher than those in the NAFLD + DMSO group (*P* < 0.05), while rhein could effectively reduce these inflammatory cytokines. However, co-treatment with AMPK-IN-3 significantly weakened this anti-inflammatory effect (*P* < 0.001).

HE staining (Fig. 7 C) revealed that liver tissues in the NAFLD + DMSO and AMPK-IN-3 groups exhibited disorganized cellular structure and extensive lipid vacuolization. In contrast, the high-dose rhein group displayed more organized hepatocyte architecture and reduced inflammatory infiltration. However, this improvement was reversed in the group treated with both rhein and AMPK-IN-3, indicating that AMPK inhibition counteracts rhein’s beneficial histological effects.

To sum up, these results provide further evidence that rhein alleviates hepatic injury and inflammation in NAFLD mice primarily through activation of the AMPK signaling pathway.

## Discussion

NAFLD is rapidly becoming one of the most prevalent manifestations of metabolic syndrome globally (Raza et al. 2021). Individuals with NAFLD face an elevated risk of their condition progressing to cirrhosis, liver failure, and hepatocellular carcinoma (Zhang et al. 2020). This study probed the roles of rhein on liver function and hepatocyte lipid metabolism in NAFLD mice by regulating the AMPK/ACC/SREBP1 signaling pathway.

We observed that the administration of rhein effectively curbed the weight gain trend in NAFLD mice, reduced the burden of kidneys, liver, and retroperitoneal fat pad weight, and effectively decreased the accumulation of lipid droplets within the liver. Simultaneously, this intervention lowered serum levels of TG and LDL-C, while elevated HDL-C levels. Notably, these positive effects were particularly prominent in the high-dose group. This suggests that rhein can effectively alleviate lipid accumulation in HFD-induced NAFLD mice and improve fat metabolism. Studies have demonstrated that rhein exerts lipid-lowering effects and also possesses antioxidant, anti-inflammatory, and insulin-sensitizing properties (Ji and Gu 2021). Further research has revealed that rhein suppresses the overexpression of C/EBP homologous protein in adipocytes and inhibits the proliferation, differentiation, and secretory activity of human preadipocytes through modulation of PPARγ signaling (Ji and Gu 2021). Moreover, in vitro studies by Qiming Xiao and his team have demonstrated that rhein effectively stimulates the expression of PPARα and its downstream proteins CPT1A and ACOX1, leading to a reduction in lipid accumulation and fibrosis progression. Their in vivo research has also revealed that rhein alleviates renal fibrosis by activating the fatty acid oxidation pathway and enhancing lipid metabolism (Xiao et al. 2022). In our study, NAFLD mice exhibited increased serum levels of ALT and AST, as well as TNF-α and IL-6, indicating significant liver function impairment and inflammatory responses. Rhein treatment significantly lowered these levels, confirming its protective effects on liver function and its capacity to suppress inflammatory mediators. These findings are consistent with those of Lingling Dong et al., who reported that rhein ameliorated ulcerative colitis by reducing intestinal inflammation and modulating the PI3K/Akt/mTOR signaling pathway and gut microbiota composition. Another study also confirmed that rhein attenuated D-GalN/LPS-induced acute liver injury by potentially altering gut microbial composition and related metabolic and gene expression pathways (Liu et al. 2022).

Research has also demonstrated that rhein alleviates diabetic NAFLD via the AMPK/SREBP1 signaling pathway, implicating lipid synthesis proteins as key mediators (Liu et al. 2024). Furthermore, the AMPK/ACC signaling axis has been shown to play a pivotal role in inhibiting lipogenesis and promoting lipolysis (Zang et al. 2023). In our study, bioinformatics analysis indicated that candidate target genes of rhein involved in NAFLD regulation were significantly enriched in the AMPK signaling pathway. Based on this, we hypothesized that rhein’s therapeutic effects are mediated by AMPK activation. Subsequent experimental validation confirmed that rhein promoted ACC phosphorylation and inhibited SREBP1 expression through activation of the AMPK pathway. Additionally, we demonstrated that rhein effectively improved lipid accumulation, liver injury, and inflammation in NAFLD mice by modulating AMPK signaling. These results align with the findings of Xiaoyan Sheng et al., who showed that targeting the liver X receptor to suppress SREBP-1c transcriptional activity and its downstream lipogenic enzymes, as well as participating in immune regulation associated with hepatic steatosis, could significantly improve NAFLD (Sheng et al. 2011).

Notably, compared with classical AMPK activators such as AICAR and Metformin, rhein not only activates AMPK but also exerts multiple biological effects, including enhancement of lipid metabolism and anti-inflammatory activity (Liang et al. 2025; Taweesap et al. 2025). For example, Metformin indirectly activates AMPK primarily by inhibiting mitochondrial complex I (Cheng et al. 2021; Ma et al. 2022), while AICAR directly stimulates AMPK by mimicking intracellular AMP elevation (Visnjic et al. 2021; Han et al. 2024), both operating via relatively single mechanisms. In contrast, rhein appears to activate AMPK through multiple upstream mechanisms, including the alleviation of oxidative stress, improvement of mitochondrial function, and suppression of inflammatory cytokines such as TNF-α. These effects collectively inhibit SREBP1 expression and enhance ACC phosphorylation, resulting in dual modulation of lipid synthesis and fatty acid oxidation. This integrated mechanism may offer synergistic advantages in NAFLD treatment, especially for patients with concurrent metabolic or inflammatory conditions. Moreover, as a naturally derived small molecule, rhein exhibits low toxicity and favorable pharmacokinetic characteristics. Future studies may explore its combination with other AMPK agonists, such as berberine or SGLT2 inhibitors, to assess potential additive or synergistic therapeutic effects for multi-targeted NAFLD management.

### Limitations

Although this study provides compelling evidence that rhein exerts hepatoprotective effects in a HFD-induced NAFLD mouse model by activating the AMPK signaling pathway—promoting ACC phosphorylation and inhibiting SREBP1 expression—several limitations should be noted. First, the study utilized only male mice, without assessing the influence of sex-based differences on NAFLD progression or the therapeutic efficacy of rhein. This is particularly relevant given the well-established gender dimorphism in NAFLD incidence and pathology observed in clinical settings. Second, while the HFD-induced mouse model is widely employed for NAFLD research, it does not fully replicate the pathological complexity of human NAFLD, particularly in patients with coexisting metabolic syndrome. This may limit the translational applicability of the findings. Third, the lack of comparison with classical AMPK agonists such as AICAR or Metformin precludes a direct evaluation of rhein’s relative efficacy and pharmacological advantages. Finally, the absence of validation using human clinical samples—such as liver tissue or serum from NAFLD patients—prevents confirmation of whether rhein modulates AMPK signaling and its downstream targets in a clinically relevant context. Future studies should incorporate both male and female animal models, develop composite diet-induced models that better resemble human NAFLD pathology, and integrate multi-omics analyses alongside human samples to enhance the clinical relevance of the findings. Additionally, the potential synergistic effects of rhein in combination with established AMPK activators warrant further investigation.

## Conclusion and clinical translation

In summary, this study demonstrates that rhein confers hepatoprotective effects in an HFD-induced NAFLD mouse model by activating the AMPK signaling pathway, thereby enhancing ACC phosphorylation and suppressing SREBP1 expression. These molecular mechanisms lead to improved hepatic lipid metabolism, reduced steatosis, mitigation of liver injury, and suppression of inflammatory responses. Identification of the AMPK/ACC/SREBP1 axis as a key regulatory pathway underlying rhein’s effects underscores its translational relevance and positions it as a promising pharmacological candidate for NAFLD therapy. From a translational perspective, this study holds significant value. First, rhein exhibits multi-target therapeutic activity, enabling simultaneous regulation of lipid metabolism, energy homeostasis, and inflammatory signaling—features well-aligned with the multifactorial pathogenesis of NAFLD. Second, the mechanistic findings suggest that rhein may be applicable not only to NAFLD but also to other metabolic-inflammatory disorders. Furthermore, as a naturally derived compound from traditional Chinese medicine, rhein demonstrates a favorable safety profile, which supports its potential for clinical development. Nevertheless, to facilitate clinical translation, further research should address current model limitations by employing disease models that more closely mimic human NAFLD, evaluating sex-specific therapeutic responses, and conducting comprehensive preclinical safety assessments. Validation of molecular mechanisms in human biospecimens and exploration of synergistic effects with existing therapeutic agents (e.g., Metformin) will be critical in accelerating the bench-to-bedside transition of rhein, ultimately contributing to safer and more effective treatment strategies for NAFLD patients.

## Supplementary Information

Supplementary Material 1.

Supplementary Material 2.

## Data Availability

The experimental data used to support the findings of this study are available from the corresponding author upon request.

## Abbreviations

AMPK: Adenosine monophosphate-activated protein kinase
ACC: Acetyl-coa carboxylase
SREBP1: Sterol regulatory element-binding protein 1
NAFLD: Non-alcoholic fatty liver disease
HFD: High-fat diet
HE: Hematoxylin and eosin
ELISA: Enzyme-linked immunosorbent assay
DMSO: Dimethyl sulfoxide
AST: Aspartate aminotransferase
TG: Triglycerides
ALT: Alanine aminotransferase
HDL-C: High-density lipoprotein cholesterol
LDL-C: Low-density lipoprotein cholesterol
IL-6: Levels of interleukin-6
TNF-α: Tumor necrosis factor-α
UC: Ulcerative colitis

## References

[CR1] Ahuja P, Ng CF, Pang BPS, Chan WS, Tse MCL, Bi X, et al. Muscle-generated BDNF (brain derived neurotrophic factor) maintains mitochondrial quality control in female mice. Autophagy. 2022;18(6):1367–84. 34689722 10.1080/15548627.2021.1985257 PMC9225428

[CR2] Cataldo I, Sarcognato S, Sacchi D, Cacciatore M, Baciorri F, Mangia A, et al. Pathology of non-alcoholic fatty liver disease. Pathologica. 2021;113(3):194–202. 34294937 10.32074/1591-951X-242 PMC8299321

[CR3] Cheng D, Xu Q, Wang Y, Li G, Sun W, Ma D, et al. Metformin attenuates silica-induced pulmonary fibrosis via AMPK signaling. J Translational Med. 2021;19(1):349. 10.1186/s12967-021-03036-5 PMC8365894 34399790

[CR4] Cui Y, Chen J, Zhang Z, Shi H, Sun W, Yi Q. The role of AMPK in macrophage metabolism, function and polarisation. J Translational Med. 2023;21(1):892. 10.1186/s12967-023-04772-6 PMC10709986 38066566

[CR5] De A, Bhagat N, Mehta M, Taneja S, Duseja A. Metabolic dysfunction-associated steatotic liver disease (MASLD) definition is better than MAFLD criteria for lean patients with NAFLD. J Hepatol. 2024;80(2):e61–2. 37558135 10.1016/j.jhep.2023.07.031

[CR6] Fang Z, Xu H, Duan J, Ruan B, Liu J, Song P, et al. Short-term Tamoxifen administration improves hepatic steatosis and glucose intolerance through JNK/MAPK in mice. Signal Transduct Target Therapy. 2023;8(1):94. 10.1038/s41392-022-01299-y PMC9981902 36864030

[CR7] Guo X, Yin X, Liu Z, Wang J. Non-Alcoholic fatty liver disease (NAFLD) pathogenesis and natural products for prevention and treatment. Int J Mol Sci. 2022;23(24):15489. 10.3390/ijms232415489 PMC9779435 36555127

[CR8] Han WM, Hong YX, Xiao GS, Wang RY, Li G. NMDARs activation regulates endothelial ferroptosis via the PP2A-AMPK-HMGB1 axis. Cell Death Discovery. 2024;10(1):34. 38233385 10.1038/s41420-023-01794-3 PMC10794209

[CR9] Ji L, Gu H. The anti-obesity effects of Rhein on improving insulin resistance (IR) and blood lipid levels are involved in Endoplasmic reticulum stress (ERs), inflammation, and oxidative stress in vivo and vitro. Bioengineered. 2021;12(1):5797–813. 34516329 10.1080/21655979.2021.1969196 PMC8806563

[CR10] Lee DS, An TH, Kim H, Jung E, Kim G, Oh SY, et al. Tcf7l2 in hepatocytes regulates de Novo lipogenesis in diet-induced non-alcoholic fatty liver disease in mice. Diabetologia. 2023;66(5):931–54. 36759348 10.1007/s00125-023-05878-8 PMC10036287

[CR11] Li GM, Chen JR, Zhang HQ, Cao XY, Sun C, Peng F, et al. Update on pharmacological activities, security, and pharmacokinetics of rhein. Evid-based Complement Alt Med: eCAM. 2021;2021:4582412. 10.1155/2021/4582412 PMC8387172 34457021

[CR12] Li R, Li Y, Yang X, Hu Y, Yu H, Li Y. Reducing VEGFB accelerates NAFLD and insulin resistance in mice via inhibiting AMPK signaling pathway. J Translational Med. 2022;20(1):341. 10.1186/s12967-022-03540-2 PMC9338666 35907871

[CR13] Li X, Xiao C, Yuan J, Chen X, Li Q, Shen F. Rhein-attenuates LPS-induced acute lung injury via targeting NFATc1/Trem2 axis. Inflamm Research: Official J Eur Histamine Res Soc [et al]. 2023;72(6):1237–55. 10.1007/s00011-023-01746-8 PMC10201049 37212865

[CR14] Liang N, Liu S, Wang Y, Ying L, Zhang K, Li H, et al. Nicotinamide mononucleotide (NMN) improves the senescence of mouse vascular smooth muscle cells induced by Ang II through activating p-AMPK/KLF4 pathway. Pharmaceuticals. 2025;18(4):553. 10.3390/ph18040553 PMC12030317 40283988

[CR15] Lin K, Yang N, Luo W, Qian JF, Zhu WW, Ye SJ, et al. Direct cardio-protection of Dapagliflozin against obesity-related cardiomyopathy via NHE1/MAPK signaling. Acta Pharmacol Sin. 2022;43(10):2624–35. 35217813 10.1038/s41401-022-00885-8 PMC9525284

[CR16] Liu F, Li Y, Li M, Wang J, Zhang Y, Du Y, et al. Study on mechanism of iridoid glycosides derivatives from fructus gardeniae in Jiangxi Province by network Pharmacology. Evidence-based Complement Altern Medicine: eCAM. 2020;2020:4062813. 10.1155/2020/4062813 PMC7336235 32714404

[CR17] Liu S, Yin R, Yang Z, Wei F, Hu J. The effects of Rhein on D-GalN/LPS-induced acute liver injury in mice: results from gut microbiome-metabolomics and host transcriptome analysis. Front Immunol. 2022;13:971409. 36389730 10.3389/fimmu.2022.971409 PMC9648667

[CR18] Liu C, Cao Q, Chen Y, Chen X, Zhu Y, Zhang Z, et al. Rhein protects retinal Muller cells from high glucose-induced injury via activating the AMPK/Sirt1/PGC-1alpha pathway. J Recept Signal Transduct Res. 2023;43(2):62–71. 37330920 10.1080/10799893.2023.2223319

[CR19] Liu Y, Sun Z, Dong R, Liu P, Zhang X, Li Y, et al. Rutin ameliorated lipid metabolism dysfunction of diabetic NAFLD via AMPK/SREBP1 pathway. Phytomedicine: Int J Phytotherapy Phytopharmacology. 2024;126:155437. 10.1016/j.phymed.2024.155437 38394735

[CR20] Ma N, Wang YK, Xu S, Ni QZ, Zheng QW, Zhu B, et al. PPDPF alleviates hepatic steatosis through Inhibition of mTOR signaling. Nat Commun. 2021;12(1):3059. 34031390 10.1038/s41467-021-23285-8 PMC8144412

[CR21] Ma T, Tian X, Zhang B, Li M, Wang Y, Yang C, et al. Low-dose Metformin targets the lysosomal AMPK pathway through PEN2. Nature. 2022;603(7899):159–65. 35197629 10.1038/s41586-022-04431-8 PMC8891018

[CR22] Oishi Y, Koike H, Kumagami N, Nakagawa Y, Araki M, Taketomi Y, et al. Macrophage SREBP1 regulates skeletal muscle regeneration. Front Immunol. 2023;14:1251784. 38259495 10.3389/fimmu.2023.1251784 PMC10800357

[CR23] Park M, Yoo JH, Lee YS, Lee HJ. Lonicera caerulea extract attenuates Non-Alcoholic fatty liver disease in free fatty Acid-Induced HepG2 hepatocytes and in high fat Diet-Fed mice. Nutrients. 2019;11(3):494. 10.3390/nu11030494 PMC6471428 30813654

[CR24] Pei R, Jiang Y, Lei G, Chen J, Liu M, Liu S. Rhein derivatives, A promising pivot? Mini Rev Med Chem. 2021;21(5):554–75. 33167832 10.2174/1389557520666201109120855

[CR25] Perakakis N, Stefanakis K, Mantzoros CS. The role of omics in the pathophysiology, diagnosis and treatment of non-alcoholic fatty liver disease. Metab Clin Exp. 2020;111S:154320. 32712221 10.1016/j.metabol.2020.154320 PMC7377759

[CR26] Raza S, Rajak S, Upadhyay A, Tewari A, Anthony Sinha R. Current treatment paradigms and emerging therapies for NAFLD/NASH. Front Biosci. 2021;26(2):206–37. 10.2741/4892 PMC7116261 33049668

[CR27] Sheng X, Wang M, Lu M, Xi B, Sheng H, Zang YQ. Rhein ameliorates fatty liver disease through negative energy balance, hepatic lipogenic regulation, and Immunomodulation in diet-induced obese mice. Am J Physiol Endocrinol Metabolism. 2011;300(5):E886–93. 10.1152/ajpendo.00332.2010 21364120

[CR28] Sheng D, Zhao S, Gao L, Zheng H, Liu W, Hou J, et al. BabaoDan attenuates high-fat diet-induced non-alcoholic fatty liver disease via activation of AMPK signaling. Cell Bioscience. 2019;9:77. 31548878 10.1186/s13578-019-0339-2 PMC6751621

[CR29] Taweesap P, Potue P, Khamseekaew J, Iampanichakul M, Jan OB, Pakdeechote P, et al. Luteolin relieves metabolic Dysfunction-Associated fatty liver disease caused by a High-Fat diet in rats through modulating the AdipoR1/AMPK/PPARgamma signaling pathway. Int J Mol Sci. 2025;26(8):3804. 10.3390/ijms26083804 PMC12028338 40332475

[CR30] Visnjic D, Lalic H, Dembitz V, Tomic B, Smoljo T. AICAr, a widely used AMPK activator with important AMPK-Independent effects: a systematic review. Cells. 2021;10(5):1095. 10.3390/cells10051095 PMC8147799 34064363

[CR31] Wei J, Zhen YZ, Cui J, He FL, Shen T, Hu G, et al. Rhein lysinate decreases inflammation and adipose infiltration in kk/hlj diabetic mice with non-alcoholic fatty liver disease. Arch Pharm Res. 2016;39(7):960–9. 27277164 10.1007/s12272-016-0770-4

[CR32] Wu X, Huang T. Recent development in acetyl-CoA carboxylase inhibitors and their potential as novel drugs. Future Med Chem. 2020;12(6):533–61. 32048880 10.4155/fmc-2019-0312

[CR33] Wu C, Bian Y, Lu B, Wang D, Azami NLB, Wei G, et al. Rhubarb free anthraquinones improved mice nonalcoholic fatty liver disease by inhibiting NLRP3 inflammasome. J Translational Med. 2022;20(1):294. 10.1186/s12967-022-03495-4 PMC9238089 35765026

[CR34] Xiao Q, Yu X, Yu X, Liu S, Jiang J, Cheng Y, et al. An integrated network Pharmacology and cell metabolomics approach to reveal the role of rhein, a novel PPARalpha agonist, against renal fibrosis by activating the PPARalpha-CPT1A axis. Phytomedicine: Int J Phytotherapy Phytopharmacology. 2022;102:154147. 10.1016/j.phymed.2022.154147 35567992

[CR35] Ye J, Tian X, Wang Q, Zheng J, Yang Y, Xu B, et al. Monkfish peptides mitigate high fat diet-Induced hepatic steatosis in mice. Mar Drugs. 2022;20(5):312. 10.3390/md20050312 PMC9147042 35621963

[CR36] Zang L, Kagotani K, Hayakawa T, Tsuji T, Okumura K, Shimada Y, et al. The hexane extract of Citrus Sphaerocarpa ameliorates visceral adiposity by regulating the PI3K/AKT/FoxO1 and AMPK/ACC signaling pathways in high-fat-diet-induced obese mice. Molecules. 2023;28(24):8026. 10.3390/molecules28248026 PMC10745821 38138517

[CR37] Zeng H, Qin H, Liao M, Zheng E, Luo X, Xiao A, et al. CD36 promotes de Novo lipogenesis in hepatocytes through INSIG2-dependent SREBP1 processing. Mol Metabolism. 2022;57:101428. 10.1016/j.molmet.2021.101428 PMC8810570 34974159

[CR38] Zhang L, She ZG, Li H, Zhang XJ. Non-alcoholic fatty liver disease: a metabolic burden promoting atherosclerosis. Clin Sci. 2020;134(13):1775–99. 10.1042/CS20200446 32677680

[CR39] Zhong H, Shi Q, Wen Q, Chen J, Li X, Ruan R, et al. Pan-cancer analysis reveals potential of FAM110A as a prognostic and immunological biomarker in human cancer. Front Immunol. 2023;14:1058627. 36923407 10.3389/fimmu.2023.1058627 PMC10008925

